# Exploring the concept inability to work fulltime in the context of work disability assessments: a qualitative study

**DOI:** 10.1186/s12889-021-11917-z

**Published:** 2021-10-13

**Authors:** Henk-Jan Boersema, Femke I. Abma, Tialda Hoekstra, Pepijn D. D. M. Roelofs, Sandra Brouwer

**Affiliations:** 1 Department of Health Sciences, Community and Occupational Medicine, University of Groningen, University Medical Center Groningen, PO Box 196, 9700 AD Groningen, The Netherlands; 2 Research Centre for Insurance Medicine AMC-UMCG-UWV-VUMC, Amsterdam, The Netherlands; 3 Dutch Social Security Institute: the Institute for Employee Benefits Schemes (UWV), Amsterdam, The Netherlands

**Keywords:** Work, Disability evaluation, Insurance medicine, Qualitative research, Working hours

## Abstract

**Background:**

In many countries inability to work fulltime is recognized as an important concept in work disability assessments. However, consensus is lacking regarding the concept and how it should be assessed. This study seeks to conceptualize and operationalize the concept of inability to work fulltime, and includes perspectives of both patients and physicians. Research questions involve identifying: 1. key elements, 2. measurable indicators, and 3. valid methods for assessing indicators of inability to work fulltime.

**Methods:**

We used a qualitative study with a thematic content analysis design to conceptualize inability to work fulltime, based on nineteen semi-structured interviews conducted among insurance and occupational health physicians, and representatives of patient organizations.

**Results:**

Inability to work fulltime is conceptualized as a complex concept which is strongly individually determined and variable due to time and underlying disease. Key dimensions of inability to work fulltime included besides the disease itself, also personal factors like psychological and lifestyle factors, as well as environmental factors related to the work situation and social context. Fatigue, cognitive impairments, and restrictions in functioning in- and outside work were reported as important measurable indicators. A combined use of self-assessment, assessment interviews, and testing, and assessment in the actual (work) setting was identified for assessing these indicators.

**Conclusion:**

Taking into account the complex and variable nature of inability to work fulltime, we found it advisable to use multiple methods and multiple time points for the assessment. Results of this study provide starting points for further research on the operationalization of inability to work fulltime in a work disability context.

## Introduction

Social security systems generally distinguish two main aims of work disability assessment: to decide about eligibility for disability benefits, and to determine what people are still able to do regarding work [1]. Included in work disability assessment is evaluation of whether a person is (un)able to work *fulltime*, i.e., whether or not employment participation is partially limited due to a health problem. As being able to work is vital for a person’s economic self-sufficiency and social standing, valid assessment is of great importance.

In many European countries, inability to work fulltime is recognized as an important concept in work disability assessments [2]; the concept includes the restricted number of hours per day or week a claimant is able to work due to a chronic disease and/or other accepted causes. A previous study comparing 16 European countries showed that a majority of them included assessment of inability to work fulltime (or restricted work endurance) as part of the work disability assessment [2]. If a person is not able to work fulltime it can be described as an inability to work fulltime. Across countries, the definition of fulltime ranged from 35 to 42 h per week [2]. Both physical and mental disorders are accepted causes of inability to work fulltime, with the most often mentioned causes being musculoskeletal diseases, mental disorders, and diseases of the circulatory system. Limited research indicates that in most countries a general deficit in energy was the most frequent indication for granting a limited work endurance [2].

The few existing studies that assess the hours a person is able to work demonstrate confusion regarding the meaning of the concept inability to work fulltime [2, 3]. First, in different countries the concept is assessed differently [2]; various methods are used to aid in assessment, the most common being clinical tests, functional capacity evaluations, and psychological tests. Second, high inter-doctor disagreements have been found on the outcome of assessing inability to work fulltime, questioning the credibility of the current assessment procedures [4–6]. In a previous study we found that, although 10 out of 13 countries use formal rules to assess inability to work, in the Netherlands only a professional guideline is used [2]. This guideline [7] describes the ability to work fulltime as the ability to work at least eight hours per day. The inconsistencies found between countries and physicians may be due to the lack of evidence-based guidelines, and of reliable and valid methods for assessing a person’s (in)ability to work fulltime, but are first and foremost due to the lack of a comprehensive conceptualization and operationalization of the concept.

Conceptualization involves formulation of clear and concise definitions: identifying the key elements, using characteristics (non-measurable key elements) and dimensions (measurable key elements). Conceptualization is followed by operationalization: making an abstract concept measurable by describing its dimensions and translating these into measurable indicators [8]. Effective conceptualizing and operationalization of the concept inability to work fulltime will thus shed light on its key elements and measurable indicators. This insight can then be used to develop methods for its assessment.

This study seeks insight regarding conceptualization and operationalization of the concept of inability to work fulltime, based, among other things, on the perspectives of both patients and physicians. To assess the concept effectively, we also want to explore its dimensions and indicators. Our specific research questions are: 1) What are the key elements (characteristics and dimensions) of inability to work fulltime?; 2) What are measurable indicators of inability to work fulltime?; and 3) Which methods can be used to assess the measurable indicators of inability to work fulltime?

## Methods

### Study design

For our study, we used qualitative interviews to explore the concept of inability to work fulltime. Qualitative research is useful for understanding complex issues, explaining people’s beliefs and behaviours, and identifying social or cultural norms [9]. To evaluate the collected data we used thematic analysis, applying elements of both phenomenology and the grounded theory approach to content analysis to conceptualize and operationalize the concept.

Dutch law required no ethical approval for this study, as participants were not subject to any intervention. All participants provided informed consent to record the interviews and publish the results, given that data were anonymized and untraceable to individuals. Participation in the study was voluntary, and participants received no incentive for participation.

### Participants

We explored the concept inability to work fulltime from the perspectives of both the patient and the physician in order to triangulate points of view from these two main stakeholders.

We invited physicians in staff and/or management positions in insurance and occupational medicine and in both public and private disability insurance, preferably with practical experience and with adequate knowledge of work disability assessment at scientific or staff levels. For the patients’ perspective, we invited representatives, expert staff members of patient organizations in the Netherlands, to participate. Patient organizations provide information, offer fellow sufferers contact, promote interests, organize activities, and support groups of specific patients not only with healthcare issues but also regarding social and employment participation. A patient organization is often established for and by patients. We purposively sampled patient representatives to include the major disease groups related to work disability (mental problems, neoplasms, and respiratory, nervous, and urogenital diseases), and to examine their experiences with the (in)ability to work fulltime. The researchers invited physicians from their own professional networks, and contacted most patient representatives through the websites of their organizations, or their professional networks. The authors approached participants by email and telephone to describe their own role, as well as the aim and context of the study.

### Data collection, interview content and procedure

Between January and September of 2014, we conducted semi-structured interviews using open-ended questions. We developed an interview guide with topics and open-ended questions to aid the interviewers and to ensure comparability of the interviews, thereby increasing reliability. We tested this script with three insurance physicians, recruited from the researcher’s own network. Based on these try-out interviews the interview guide was fine-tuned, using more open questions. We chose to interview physicians and patient representatives to acquire data on (the assessment of) inability to work fulltime from the perspective of the key participants in the disability assessment interview.

The final interview guide addressed the following major topics: 1) the concept of inability to work fulltime and its characteristics; 2) dimensions of inability to work fulltime; 3) indicators for measuring the dimensions of inability to work fulltime (signs and symptoms of the concept and its dimensions); and 4) methods to assess indicators of inability to work fulltime. Subtopics included: what is ‘normal ability to work fulltime’, or the maximum number of hours a person can work; disease specific aspects related to variability of inability to work fulltime; the best method to assess indicators of inability to work fulltime; and experience with assessing inability to work fulltime. To explore these topics more deeply, we asked further clarifying questions. Of the 19 interviews, 18 were conducted by two interviewers (HJB and senior researcher and insurance physician BC or research assistant JS [*more information about the research team members can be found under Acknowledgements]);* one interview was conducted by the first author only (HJB). We conducted all interviews in the participants’ first language (Dutch), during single sessions of 45–90 min; all were audio-recorded. We made no additional field notes. We interviewed most participants at their own preferred locations, and two by telephone; no other persons were present during the interviews. We transcribed all interviews verbatim. We did not present transcriptions to the participants for their comments, but presented and discussed our interpretations of the data at professional meetings with researchers, professionals, and policymakers in the field of work and health.

### Data analysis

The first author verified all transcripts. We used thematic analysis to analyse the collected data [10]. We used an inductive approach to analyse the data, starting with line-by-line coding of the transcripts, using Atlas-ti (version 7.5.18) computer software. During this open coding process, we developed an initial list with codes. All data were coded by the main researcher, HJB, and two members of the research team (BC and FA), and codes were ultimately grouped and combined into subthemes in an iterative manner. We held weekly meetings to discuss disagreements in the coding and grouping processes, until reaching consensus. The last stage consisted of discussions among members of the research team (HJB, BC, FA, SB, PR, TH) until consensus was reached on the final themes. Data saturation was not the aim of this study, as we wanted to explore themes among representatives from major disease groups. All members of the research team work at the University Medical Center Groningen and are affiliated with the Research Center for Insurance Medicine. The first author, HJB, is an insurance physician and PhD candidate; FA has a background in work and organizational psychology; TH and SB have backgrounds in health sciences; and PR has a background in health sciences and occupational physiotherapy. FA, PR and SB have PhDs in the domain of work and health research, and are experienced in conducting qualitative research. Additionally, BC, who played an important role in analyzing the data, was an insurance physician and a senior researcher at the Research Center for Insurance Medicine, with a PhD in work and health. The mixed backgrounds of the team members enriched the analysis by introducing different perspectives. Analyses were influenced by the first author’s experience in conducting actual work disability assessments, and his extensive knowledge on the topic inability to work fulltime.

We summarized and searched the texts underlying the themes and codes to find quotes that best illustrate the views and experiences of the interviewees. Quotes from interviewees were selected by two authors (HJB, FA), translated into English by a professional translator, and discussed with all co-authors. To indicate the diversity of opinions while maintaining anonymity, we indicate quotes from physicians with Ph1-Ph10, and from patient representatives with Pa1-Pa9.

In the final iteration, we formed a conceptualization based on emerging themes describing the key elements. We used these key elements to operationalize the concept into relevant characteristics, dimensions and measurable indicators, and inventory methods for assessing these indicators. Although the interview guide contained no questions regarding the International Classification of Functioning, Disability and Health (ICF) [11], we were able to identify and categorize responses to this framework. Other sources of categorization were national guidelines on prescribing adequate, and/or reducing, working hours [7, 12]. Within the research team we discussed characteristics, dimensions and indicators to compose an overview of the concept inability to work fulltime.

## Results

### Participant characteristics

We initially invited 33 persons (13 physicians and 20 patient representatives) for interviews, 19 of whom (ten physicians and nine representatives of patient organizations) agreed to participate. Reasons for refraining from participation varied: lack of time, illness, insufficient expertise, or the topic or interview did not fit with the scope of the organization.

In our final group, seven out of ten physicians were insurance physicians: four working in public disability insurance (Ph1–4), and three working in private disability insurance (Ph5–7). Three participants were occupational health physicians (Ph8–10). Nine physicians were male, and five had obtained a PhD-degree. The nine staff members from patient organizations represented patients with five disabling chronic diseases (mental and behavioral conditions (*n* = 3) Pa1–3, diseases of the nervous system (*n* = 3) Pa4–6, genitourinary system disorders (*n* = 1) Pa7, neoplasms (*n* = 1) Pa8, and diseases of the respiratory system (*n* = 1) Pa9). All subjects had received higher education, most at university level; six were female. All worked as project manager, (senior) staff member, or advisor.

### Main findings

Overall, findings from the two stakeholder groups corresponded, with only a slight difference in point of view on inability to work fulltime. In the interviews, discussion of the key elements (dimensions and characteristics), the measurable indicators, and the related assessment methods was intertwined. An overview of the terminology and main findings is presented in Table 1. Patient representatives tended to describe the inability to work fulltime from a more holistic perspective, while physicians, and especially insurance physicians, used a more narrow bio-medical perspective**.**

**Table 1:** Conceptualization and operationalization of the concept ‘inability to work full time’

**Conceptualization (identifying key elements)**	
*Characteristics*	Inability to work normal working hours
	Variability of inability to work fulltime: - due to time - due to underlying disease
*Dimensions*	
	Disease and personal factors (i.e. psychological and lifestyle factors)
	Environmental factors (i.e. work-related and social factors, and norms)
**Operationalization (measurable indicators)**	
*Indicators*	
	Fatigue
	Cognitive impairments
	Restrictions in functioning in- and outside work
*Assessment methods*	
	Self-assessment
	Assessment interviews
	Functional testing
	Assessment in the actual work setting

### Conceptualization of inability to work fulltime

#### Characteristics of inability to work fulltime

Most participants found inability to work fulltime a complex concept to operationalize. It describes the inability of a person to work normal working hours, i.e. not able to work a normal number of hours per day and per week. Important characteristics of inability to work fulltime are that it is strongly individually determined and is variable. One patient representative stated when asked; *“What do you consider a normal work-time capacity?”: “that, of course, varies from person to person”* Pa8). A physician stated: *“the work capacity, that is different for each person”* (Ph7). Additionally, two aspects were described characterizing the variable nature of inability to work fulltime**:** variability due to time, and variability due to the underlying disease.

##### Variability due to time

Inability to work fulltime varies over time due to many different factors. As one physician stated: “*We are not machines. We are influenced by all sorts of things happening in and outside ourselves over time. That varies all the time”* (Ph3). Another physician stated: *“When you’re talking about work ability in terms of a social norm, I think it varies over time. Nowadays we expect other things from people than twenty years ago”* (Ph1).

Both physicians and patient representatives mentioned that with age, people have a reduced capacity to bear physical and cognitive strain, need more recovery time, and are less resilient. However, physicians also described a learning curve over time, involving the development of more (cognitive) skills that may compensate for this reduced physical capacity; as one physician stated: *“The physical capacities may decline somewhat over the years, but you can make up for that with things like increased skills”* (Ph7).

##### Variability due to underlying disease

Participants mentioned that variation in severity and complaints, the effect of treatment and training, and personal and external factors may affect a person’s ability to work fulltime. The type of disease was often mentioned as a variable factor related to, and a potential indicator of, the impaired ability to perform working activities; examples were severe heart failure and chronic renal insufficiency. However, they stressed that not only having the disease (the diagnosis) itself causes inability to work fulltime, but also the course of the disease. Physicians remarked, “*Even some people with depression are able to work*” (Ph1), and “*We all know that a well-known feature of all kinds of depressive disorders is that they fluctuate”* (Ph3).

Additionally, most participants mentioned treatment and rehabilitation as factors influencing the number of hours a person can work. For example, cancer treatments and time-consuming kidney dialyses were mentioned as significant barriers to being able to work fulltime. However, cancer rehabilitation, sports, cognitive training, and stepwise functional recovery were mentioned as factors that positively influence inability to work fulltime, regardless of the person’s diagnosis. A physician stated: *“Well, work ability varies with the clinical picture, the health condition, whether the condition is active, and whether there are treatment options now or in the future”* (Ph2).

#### Dimensions of inability to work fulltime

##### Disease and personal factors

Besides the type of disease, several personal factors were mentioned as key dimensions of the inability to work fulltime. Physicians reported further psychological factors, such as a person’s (in)ability to cope, as well as motivations and orientation in life, as important aspects that influence the number of hours a person can work. Patient representatives mentioned an improved lifestyle (e.g., smoking cessation, more exercise), positive orientation and goals in life, the choice to work self-employed, having self-confidence, and coping with the disease to be of influence. One physician said: *“Some people just get hung up on it; others don’t*” (Ph3). One patient representative stated: *“Some people just want to achieve a higher ability to work because it has to do with certain personal life goals*” (Pa9).

##### Environmental factors

As environmental factors, physicians mentioned work-related factors (e.g., workload, work content, work autonomy, commuting time) and workplace factors (e.g., facilities, noise, light, climate). Patient representatives added organizational policies and practices, social support, job control and job fit, conflicts at work, discrimination, and re-organization as factors associated with the ability to work fulltime. One physician said, “*The moment you create more possibilities at work, people have the ability to make a positive contribution to work, even at higher ages”* (Ph9). A patient representative said, *“All circumstances at work, and whether or not you are satisfied with them, play a very important role in your work capacity”* (Pa2).

Regarding social factors, we found that workers’ social situations can impact the number of hours they are able to work. A person’s household, family obligations, family concerns, problems and worries, may negatively influence the ability to work a certain number of hours per day or per week. However, family support can also have a positive effect. A physician said, *“When you have big problems in your private life, you can be physically able to work, but your true ability to work and your productivity will be lower as long as these issues are not resolved”* (Ph9). A patient representative said, “*When you have a good partner, good support and feel well, you are better able to cope with your limitations”* (Pa6)*.*

Further, most participants stated that societal norms strongly influence what is generally considered to be normal. Both physicians and patient representatives considered fulltime working as normal, but the number of hours per day and per week may differ, depending on societal norms. These norms can be based on legal and collective arrangements between employers and employees regarding working conditions, on policies within companies, and on insights within social groups. A physician said, *“What is expected of a worker is based on legal or social norms. Apparently, a Dutch fulltime employee is legally required to work 40 or 38 hours, depending on the labor agreement, or fewer hours, depending on the employment contract. But that doesn’t say anything about his physical ability”* (Ph5). A patient representative said, *“I think that most people are able to work between 30 to 40/50 hours (per week), but it strongly depends on where you come from and on your upbringing”* (Pa3). Participants generally agreed that every person has his or her own maximum of hours that he/she can work, and stated that it is impossible to prescribe a universal maximum of working hours. A patient representative stated, *“I think the maximum amount is very personal and very much dependent on the sort of work you do. I don’t think there is an upper limit that applies to everyone”* (Pa8). Physicians stated that the maximal number of hours a person can work per day or per week may differ from person to person, ranging from 9 to 12 h per day and from 55 to 80 h per week. According to the physicians, the upper limit is influenced not only by health status, but also by personal factors (physiology, coping abilities, motivation, training), and environmental factors (individual workload, safety requirements, home situation). Frequently exceeding one’s maximum may lead to long-term health complaints and negative health effects, indicating a need to recover from physical and mental work efforts for a shorter or longer period of time. A physician stated, “*Research shows that people make more mistakes, get tired and have more problems concentrating if they work longer than nine consecutive hours without a break”* (Ph9). A patient representative said, *“In earlier days, and nowadays in some countries, people worked from sunrise to sunset, and then went to sleep. That’s exhausting, and that’s why these people didn’t get very old”* (Pa3).

### Operationalization of inability to work fulltime into measurable indicators

#### Indicators of inability to work fulltime

We found three relevant measurable indicators to assess inability to work fulltime: fatigue, cognitive impairments, and problems in functioning in- and outside work. Patient representatives of patients with somatic diseases mentioned more physical indicators (“*slow recovery”, “specific disease-related complaints like pain and dyspnea”*) while those representing patients with mental disease mentioned more cognitive indicators (*“execution of complex tasks”, “overview of situations”, “coping with emotions”,* and *“environmental stimuli”*).

##### Fatigue

Fatigue was reported as an important indicator of inability to work fulltime. Patient representatives stated that people with inability to work fulltime *“lack the energy”* (Pa8, Pa1, Pa7), and *“run into all kinds of barriers”* (Pa8, Pa4). Physicians stated that these people *“feel unable to work the whole day”* (Ph8), that *“they can’t accomplish anything anymore after six hours of work”* (Ph8).

##### Cognitive impairments

Physicians stated that people with inability to work fulltime *“can’t cope any longer*” (with a full day’s work) (Ph5), that they *“need more time to understand things”* (Ph2). Participants also mentioned that people with inability to work fulltime have problems with cognitive and complex tasks, stating that they *“forget”* (Pa7), *“make mistakes”* (Ph1, Ph2, Ph4, Ph10), *“have no overview”* (Pa4, Pa2, Pa3), and “*have fewer problem-solving abilities”* (Pa5). Some also mentioned emotional complaints as indicators of inability to work fulltime, such as *“irritability”* (Pa4, Pa5), *“less able to cope with conflicts”* (Pa4), and *“mental decompensation”* (Pa5).

##### Restrictions in functioning in- and outside work

Most participants reported that people who cannot work fulltime have problems with functioning both in- and outside work. They emphasized the importance of having sufficient time to recover from work, and balancing work with other activities like household tasks, self-care, and social activities.

For example *“doing less”* (Ph6), *“needing a power nap”* (Ph9), *“being unable to do anything in the evening hours after work”* (Ph3), *“not being able to get out of bed”* (Ph7), *“going to sleep during the day”* (Pa4, Pa3), *“[making] mistakes in their work”* (Ph9), “*function[ing] less well at work when they continue to work longer”* (Ph2), *“[being unable to] visit friends anymore in the evening”* (Pa7), and *“not [being] able to go out anymore or do sports”* (Pa4).

### Assessment methods of inability to work fulltime

Quantifying the number of hours per day a person can work is seen as an enormous challenge. As one physician indicated, “*It is relatively easy to determine that someone is unable to work fulltime, but when it comes to assessing the level of inability to work fulltime we are just swimming”* (Ph4).

After we explored how best to assess the indicators of inability to work fulltime four methods emerged: self-assessment, assessment interviews, functional testing (e.g., Functional Capacity Evaluation (FCE), psychological tests and ergometry [e.g., exertion test and VO2max-determination]), and assessment in the actual work setting. Although there was no consensus about a single best method, most participants found it insufficient to use only one instrument.

Self-assessment methods alone were not regarded as a suitable measure. Patient representatives pointed out that “*people with certain disorders, like depression*, *may have trouble realizing their own limitations”* (Pa1). Physicians also stated that a client’s own estimation of functional impairments, activity limitations, and participation restrictions may need to be complemented with additional information, such as that provided by a semi-structured assessment interview.

Although most physicians considered an assessment interview to be an important method, especially in combination with other methods, patient representatives found such interviews invalid. They considered the method too simplistic; as one patient representative (Pa3) stated, “*the simple conversation at the social security institute doesn’t work*”. They declared that the assessment interview should also include *“examples of functioning and daily activities, information from treating physicians, and checking for inconsistencies”* (Ph5), *as well as “recovery after exertion, the personality of the client, and the psychosocial situation”* (Pa7), and could be supplemented with *“speaking with people next to clients, like significant others, employers or mentors”* (Pa3), and gathering *“information about what happened before, in the first two years of sick leave*” (Pa4).

Most participants (both physicians and patient representatives) mentioned the value of clinical tests, separately or in combination with other methods, as methods for assessing indicators of inability to work fulltime, such as fatigue and cognitive problems, and not just inability to work fulltime in itself. As a physician explained, “*Testing with neuropsychological assessment and ergometry (exertion-tests and VO2max-determination) contributes to the assessment of inability to work fulltime, but is not the final answer*” (Ph6); this applies especially to certain disorders or conditions like traumatic brain injury, and heart and lung diseases.

Both physicians and patient representatives regarded trial placements and observation at work as appropriate ways to discover the extent of a person’s capacity to work. This trial and error setting makes it possible to test and observe indicators of inability to work fulltime. As a patient representative stated, “*Observe if someone makes mistakes or takes rest during trial placement*” (Pa5). Participants indicated a need for repeated observations and long-term follow-up, suggesting periods from six weeks to three months.

## Discussion

In this study we aimed to conceptualize and operationalize the concept inability to work fulltime by interviewing physicians and patient representatives and analyzing their answers. Our results show that inability to work fulltime describes the inability to work normal working hours and is considered a complex concept to operationalize, as it is strongly individually determined and variable. The underlying disease and changes in the situation over time make the concept variable. Moreover, we found that key dimensions of inability to work fulltime included not only the disease itself, but also personal factors like lifestyle and psychological components, as well as environmental factors related to the work situation and conceptions regarding what constitutes a ‘normal’ number of working hours. Fatigue, cognitive impairments, and restrictions in functioning in- and outside work were reported as important measurable indicators of inability to work fulltime. To assess this inability, participants regarded assessment interviews, testing, and evaluation in the actual work setting as the most suitable methods for measuring indicators, and expressed a preference for their combined use. They also mentioned the importance of repeated assessment, given the longitudinal and variable nature of inability to work fulltime. Self-assessment methods alone were not considered suitable. In all, the results of this study provide insight into the key elements (characteristics and dimensions) of inability to work fulltime, some important measurable indicators, and methods that can be used for assessment. Our results thus contribute to more evidence-based work disability assessments.

Our findings indicate that the inability of workers to work fulltime, due to disabling chronic health conditions and/or other causes, should be treated as a complex set of personal and environmental factors, and is variable. This aligns with the conclusions of other research in this area, that the inability to work fulltime has a complex character. This underlines the complexities of work disability assessments in general [13, 14]. Measuring of complex concepts often requires multiple measures and methods. Our results correspond with the ICF-model and the dimensions of the biopsychosocial model currently applied in most work disability settings, and confirm that using solely a medical perspective is too narrow [15].

Operationalization of the concept inability to work fulltime revealed a broad perspective involving the disease, personal factors, and environmental factors, and emphasized the importance of the context of the individual. In this context, his/her career is significantly influenced by societal norms, cultural aspects, and policies regarding accepted norms for working fulltime [16, 17]. Although in this study we did not further explore this normative aspect, for further assessing and operationalizing inability to work fulltime it is vital to take this aspect into account. For example, in a more individualistic culture [18] like the Netherlands, the government has recently been advised to allow workers autonomy in determining the number of hours they work in order to maintain their work-life balance [19].

The reported measurable indicators of inability to work fulltime can be used as signals to assess the number of hours a person is able to work. They are crucial starting points to help assessors to unravel the question of reduced ability to work fulltime. However, some indicators are not necessarily only preconditions of an inability to work fulltime, as they can also be described as generic and frequently occurring expressions of disease. For example, although fatigue can be caused by a disease or treatment, and be a reason to stop working or reduce tasks, it can also be a response to working too many hours or having too great a workload. Our results are in line with the Dutch guideline [7] which mentions fatigue and cognitive impairments as aspects requiring special attention because they may be related to loss of energy and increased need for recovery.

Several validated self-report instruments are available to measure the reported indicators, such as questionnaires on fatigue or energy deficits [20, 21], pain [22], cognitive impairments [23], and functioning [24–26]. Data from these questionnaires can be supplemented with more objective measurements, as from tests like the Psychomotor Vigilance Test [27]; ActiGraph [28]; observation, for example during work and daily life [29] – combined with an assessment interview (as it was mentioned that self-assessment alone was not sufficient, because of the uncertainty regarding the use of this approach alone). This conclusion is in line with that of previous research, which raised some questions about the validity of self-reported disability measures for quantifying actual function in work disability settings [13, 30–32]. When used in combination, the above-mentioned measures can help to estimate and quantify the role of the specified indicators and strengthen the credibility of assessment outcomes. Additionally, as participants also mentioned, information from significant others like treating physicians, partners, employers, and occupational health physicians, as well as information based on previous assessments and re-integration during the period of sick leave, could be used. This is also in line with the Dutch guideline, which advises combining data from the assessment interview with additional data from tests like exercise tests and FCE, findings from significant others, and information about the subject’s personal and social situation in order to assess the inability to work fulltime [7]. Rugulies [13] discussed the advantages and disadvantages of using, among others, self-administered questionnaires and observer-based assessments, and also advised using a combination of methods. Repeated assessment should also be considered, given the longitudinal and variable aspects/dimensions of inability to work fulltime.

Further research is needed to evaluate the measurement properties of the different assessment methods and their combinations.

### Strengths and limitations

To our knowledge, this is the first study to follow a multi-perspective approach to conceptualize and operationalize the concept inability to work fulltime as part of disability assessment. It is promising both physicians and patient representatives made similar observations. In addition, the study included a wide variety of physicians and patient representatives, thereby providing broader insights into the characteristics, dimensions, indicators and methods of assessing inability to work fulltime.

This study also has some limitations. First of all, the study included only representatives of patient organizations. Although some of them are patients themselves, it is their job to lobby for the interests of their organizations; including a wider variety of patients might have produced different data. Further, the physicians were more often male, and representatives of patient organizations were more often female, but we expect that this did not influence our study results, as congruent findings were found from both perspectives. A second limitation may be some educational bias, as our participants had all received higher education; we may thus have missed relevant responses from people with less education regarding inability to work fulltime. This may reduce the generalizability of our study findings. Finally, our interviews took place in 2014; nevertheless, we are convinced that our data are still valid, as the practice of assessment of inability to work fulltime has not changed since that time.

### Practical implications

Although our exploratory work cannot deliver a totally clear definition and operationalization of inability to work fulltime, it clearly indicates areas worthy of future research and practice. The complex nature of inability to work fulltime requires comprehensive assessment methods, combining subjective and objective measures, to allow for a grasp of the multiple indicators related to the concept. This conclusion corresponds with findings in previous research on measuring complex concepts [8, 13, 14], and newly developed assessment measures in work disability settings [33]. Internal and external factors make a person’s inability to work fulltime variable, and therefore difficult to assess, especially at a single time point. To address such fluctuations, when assessing work disability we recommend measuring repeatedly, and over longer periods of time.

## Conclusion

Inability to work fulltime is considered a complex concept to operationalize. It is strongly individually determined and variable, and depends not only on disease and personal factors, but also on environmental factors. We found three important measurable indicators: fatigue, cognitive impairments, and restrictions in functioning in- and outside work. To assess inability to work fulltime, participants mentioned assessment interviews, testing, and assessment in the actual work setting as the most suitable methods, and expressed a preference for the use of combined methods; they regarded self-assessment methods alone as inadequate. Taking into account the complexity of inability to work fulltime, and its possible variation, we would thus recommend using multiple methods, and at multiple time points. The results of this study provide starting points for further research on the operationalization of inability to work fulltime in the work disability context, and contribute to more credible work disability assessments.

## Data Availability

In this qualitative study, all relevant interview data involving written transcripts of audio recordings by respondents can be found within the main text of the manuscript. The corresponding author is available for questions regarding the data.

## Abbreviations

ICF: International Classification of Functioning, Disability and Health
FCE: Functional Capacity Evaluation
